# FASTEN IIoT: An Open Real-Time Platform for Vertical, Horizontal and End-To-End Integration

**DOI:** 10.3390/s20195499

**Published:** 2020-09-25

**Authors:** Felipe S. Costa, Silvia M. Nassar, Sergio Gusmeroli, Ralph Schultz, André G. S. Conceição, Miguel Xavier, Fabiano Hessel, Mario A. R. Dantas

**Affiliations:** 1 Department of Informatics and Statistic (INE), Federal University of Santa Catarina (UFSC), Florianopolis 88040-900, Brazil; silvia.nassar@ufsc.br; 2 Politecnico di Milano, Department of Management, Economics and Industrial Engineering DIG, Polytechnic Milano, 20133 Milano, Italy; sergio.gusmeroli@polimi.it; 3 PACE/TXT, 20126 Berlin, Germany; ralph.schultz@pace.de; 4 Department of Electrical Engineering (DEE), Federal University of Bahia—UFBA, Salvador 40210-630, Brazil; andre.gustavo@ufba.br; 5 Faculty of Informatics, Pontifical Catholic University of Rio Grande do Sul (PUC-RS), Porto Alegre 90619-900, Brazil; miguel.xavier@pucrs.br (M.X.); fabiano.hessel@pucrs.br (F.H.); 6 Department of Computer Science (DCC), Federal University of Juiz de Fora (UFJF), Juiz de Fora 36036-330, Brazil; mario.dantas@ice.ufjf.br; 7 INESC P&D, Santos 11055-300, Brazil

**Keywords:** industry 4.0, IIoT, digital transformation, open source, middleware, platform, FASTEN

## Abstract

The Industry 4.0 paradigm, since its initial conception in Germany in 2011, has extended its scope and adoption to a broader set of technologies. It is being considered as the most vital mechanism in the production systems lifecycle. It is the key element in the digital transformation of manufacturing industry all over the world. This scenario imposes a set of major unprecedented challenges which require to be overcome. In order to enable integration in horizontal, vertical, and end-to-end formats, one of the most critical aspects of this digital transformation process consists of effectively coupling digital integrated service/products business models with additive manufacturing processes. This integration is based upon advanced AI-based tools for decentralized decision-making and for secure and trusted data sharing in the global value. This paper presents the FASTEN IIoT Platform, which targets to provide a flexible, configurable, and open solution. The platform acts as an interface between the shop floor and the industry 4.0 advanced applications and solutions. Examples of these efforts comprise management, forecasting, optimization, and simulation, by harmonizing the heterogeneous characteristics of the data sources involved while meeting real-time requirements.

## 1. Introduction

Starting from its initial conception of the full adoption of Cyber-Physical Systems (CPS) [1] in production, the fourth industrial revolution has emerged in Germany in 2011 [2]. Now it is a global innovation paradigm for the manufacturing industry worldwide, with several Industry 4.0 initiatives being developed in the most industrialized countries. CPS are engineered systems that are built from and depend upon, seamless integration of physical and computational components. Advances in CPS will enable capability, adaptability, scalability, resiliency, safety, security, and usability that will expand the horizons of these critical systems [3,4,5,6,7].

In parallel, the expansion of the Internet and the advances in miniaturization, speed, power, and mobility, have led to the widespread use of networks and information technologies (IT) in all economic sectors. Leading the construction of what we now call as Internet of Things (IoT), that could be understood as “dynamic global network infrastructure with self-configuring capabilities based on standard and interoperable communication protocols where physical and virtual ‘things’ have identities, physical attributes, and virtual personalities and use intelligent interfaces, and are seamlessly integrated into the information network” [6,8,9].

These two technologies have provided great changes in the interaction with systems and with information: CPS technologies are transforming the way people interact with engineered systems, just as the Internet has transformed the way people interact with information. Following this trend, the concept of the full adoption of CPS in production, the Industry 4.0 paradigm, expanded its approach and utilization to a larger set of technologies and the main processes of production systems. In all Industry 4.0 environments (Industrial Internet of Things [10,11], Additive Manufacturing [12], and Robotics for example) we are talking about top priority challenges.

In this scenario, the intelligent manufacturing systems created by Industry 4.0 can be operated globally and managed centrally, integrating them into the CPS. As a result, the communication and networking technologies that connect smart factories in the CPS environment and the security technologies that protect connections will play a critical role in this area. However, considering that this environment has big data characteristics, it is necessary that a large volume of data transactions be sent to the CPS for analysis. Thus, research in this area should address the development of network protocols optimized for transferring large volumes of data to cyber platforms and security technologies like encryption and authentication protocols to protect data transformation [6].

Therefore, the Industry 4.0 paradigm involves the integration of massively deployed smart computing and network technologies in industrial production and manufacturing settings for the purposes of automation, reliability, and control, implicating the development of an Industrial Internet of Things (IIoT). Specifically, IIoT is devoted to adopting the IoT to enable the interconnection of anything, anywhere, and at any time in the manufacturing system context to improve the productivity, efficiency, safety, and intelligence [13].

In line with the Industry 4.0 new paradigm [14] and an EU-Brazil collaboration program, the Flexible and Autonomous Manufacturing Systems for Custom-Designed Products (FASTEN), a project funded by the EU Horizon 2020 program, intends to be a key enabler of the full adoption of IoT technologies in digital manufacturing businesses. This target will be achieved by defining common goals for Europe and Brazil, and through the development of the “FASTEN framework” on top of available standards, hardware, and open source software. The demonstration of the technologies will be performed in two pilot cases: one of them specified by Embraer Portugal (EMBPT), in Portugal, and the other by ThyssenKrupp (TSK) in Brazil.

This paper is organized as follows. Section 2 presents the motivations for the development of the FASTEN project. The initial application scenario (pilots) with the definition of the research problem, including the pilots’ demands, the requirements to be met by the solution, a general description of the proposal, and finally the design and implementation of the IIoT platform. The related papers are presented in Section 3. Section 4 presents the use cases for the platform. Section 5 highlights a discussion on the real-time approach of the proposal, the main scientific contributions of the research work, as well as the contributions to each pilot. Section 6 presents the conclusions and plans for future work.

## 2. Motivations, Problem, Requirements, and Proposed Definition

### 2.1. Motivation

In order to cope with an increasing demand diversity, products with shorter life cycles, and low volumes per order, manufacturing companies require flexible solutions, capable to effectively manufacture and fast deliver more personalized products.

Factory automation systems have always been complex distributed systems and have traditionally anticipated the requirement to integrate, utilize, and control devices (e.g., machinery). Therefore, factory automation can be considered as a domain where IoT has always played a significant role. What is currently changing is the increase of intelligence in the devices, the increase in the number of smart devices, the extension of device visibility outside the factory walls (e.g., extended supply chain, distributed or virtual manufacturing), and the raising role of commodities devices and Internet standards in manufacturing. All these changes, which are not exclusive to the manufacturing domain, are impacting the following areas: (1) technologies for smart devices connection and integration; (2) access control management; (3) distributed and reactive computing; (4) semantics and IoT. On the first point, on the one hand, we need technologies to improve connectivity of smart devices (especially the ones with constrained resources), on the other hand, platforms that can easily integrate, and manage, potentially huge numbers of smart devices and efficiently collect and dispatch data from them (or to them).

In this sense, this study has as main objective to propose a solution that can provide a complete, secure, scalable, and real-time digital integration at the horizontal, vertical, and end-to-end levels in an Industry 4.0 environment using open source solutions.

### 2.2. Scenario

TSK and EMBPT are two large companies that seek to permanently overcome challenges such as those described above. TSK supply, from Brazil to South American countries, many components to be used during the maintenance services of their elevators and escalators. In this case, aspects like level of service, time and cost to manufacture unique parts, and inventory costs can be dramatically improved by incorporating IoT technologies, both in manufacturing and in the material handling processes. The main objective here is to implement a system that provides immediate part availability, thus improving maintenance operations. For this purpose, operators (clients) will be able to place orders, and afterward the system will readjust itself in real-time to meet that specific demand. 3D printing technologies allow for complex pieces to be produced without additional setup times, and therefore the predictive simulation-optimization tool will be able to decide on the best strategy (layout rearrangement, scheduling, etc.) to achieve production requirements, with minimal costs.

EMBPT wants to increase its warehousing flexibility, to be able to cope with changing demands. This implies a new approach based on innovative robotic and stock management solutions. Advanced robots and smarter localization strategies, IoT, and machine learning technologies should be used to improve material storage, location, retrieval, and grouping, to maximize automation, flexibility, and responsiveness. Furthermore, the possibility to introduce more customization capabilities (with additive manufacturing solutions) to some parts of the executive jets produced in Portugal should be assessed, as customers in this market often request superior levels of personalization for the interior components, like seat pieces and other accessories. Full incorporation of all stored items into the IoT cloud will provide the necessary information for the predictive and prescriptive real-time simulation-optimization to track these items and to assist the system in directing the multipurpose robots, for optimized storing and retrieval [15,16]. This will be done using advanced machine learning and real-time plant monitoring, without requiring rigid indexing of moving parts inside the warehouses.

Therefore, both TSK and EMBP project pilots can benefit from the FASTEN framework that combines smart sensor and process control, simulation-optimization tools, additive and robot manufacturing technologies, to design a fully automated and networked cyber-physical manufacturing system.

For this purpose and in contrast to conventional manufacturing systems, FASTEN will develop a fully connected and scalable manufacturing system, integrating robotic, automation, simulation, as well as optimization and prescriptive analytics technologies, to produce one-of-a-kind customer designs. The FASTEN framework will be the basis for a system developed to support smart manufacturing [17], to help decision making, and to act in real-time, in the context of mass customization, providing complete integration at the horizontal, vertical, and end-to-end levels. The horizontal is the integration of IT systems used in the different stages of the manufacturing and business planning processes inside a company or between different companies. The vertical is the integration of IT systems at the different hierarchical levels (sensor, manufacturing, execution, etc.). Finally, end-to-end integration occurs throughout the engineering process, so that the digital world and the real world are integrated throughout the value chain of a product and in different companies, as well as incorporating customer requirements [18].

**Figure 1:**
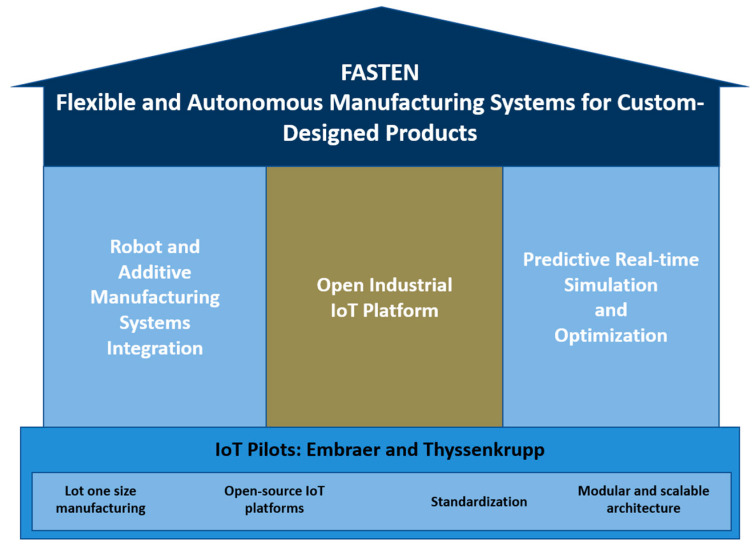
Flexible and Autonomous Manufacturing Systems for Custom-Designed Products (FASTEN) framework supported by three pillars and the pilots.

The framework will be designed following the reference architecture model RAMI 4.0 [19], supported by three pillars associated with specific research and innovation components, supported by the Portuguese and Brazilian IoT pilots represented in Figure 1. Full integration of control and operational decisions is critical to enhance the economic performance of manufacturing systems. In FASTEN this integration will be accomplished by fitting together the developments of pillar 1 (a flexible and scalable manufacturing system using robotic and additive manufacturing technologies) with the developments that result from pillar 2 (an open IoT platform for manufacturing execution and customer services), and with the results derived from pillar 3 (a data analytics application for predictive and prescriptive analysis of the machinery performance and customer services for digital manufacturing).

This paper describes the efforts involved in developing an open Industrial IoT platform and how these efforts were able to meet the requirements of the pilot cases with respect to an Industrial IoT platform. In this project, technologies were used to allow higher levels of connectivity and greater integration between robotic technology and additive manufacturing units represented by pillar 2 of the FASTEN framework. The open industrial IoT platform was developed to allow real-time vertical and horizontal integration of the manufacturing system, where vertical integration will ensure that data, events, and information flow seamlessly from the real world to the digital world and vice versa (sensing-actuating loop), within the same manufacturing system. Horizontal integration, in turn, aims to provide open standard APIs to access and manipulate heterogeneous data sources originating from different manufacturing systems, in end-to-end value streams. The FASTEN platform will allow the deployment of continuous feedback between the Physical and the Cyber Manufacturing System (i.e., the decision-making tools). Thus, emphasis will be given to the task of collecting and managing considerable amounts of distributed data, where sample rates of quite different orders (seconds, minutes, hours, days, and weeks) must be handled at the different decision levels.

The FASTEN platform intends to meet the following requirements, adapted from the proposal of [20]:

- Distributivity: the platform must be able to run on independent computers, connected via a network, which can be seen as a single and consistent system.
- Interoperability: the platform must be able to connect devices and systems, from different suppliers. Application protocols and message standards are considered.
- Scalability: the platform must be prepared to handle an increasing volume of work in a uniform way, that is, be prepared to grow.
- Security: a huge network, with many different devices, in an environment where any malicious intervention can cause huge losses, security becomes a key factor. The platform must provide mechanisms to prevent malicious interventions that may pose risks to information security.
- Resource control: the platform must be able to deal with the flood of IoT data from heterogeneous resources and services, using mechanisms like indexing, discovering, and ranking these devices [21], in order to provide a list of the most reliable devices for the execution of a certain task.
- Flexibility: the solution must accommodate the specific requirements of each company, instead of being a ready and unique solution for all customers.
- Real-time data processing: the solution must provide resources to support services with runtime restrictions.
- Persistence: the platform must allow database managers capable of handling data of the most diverse types, including streaming data.

### 2.3. FASTEN IIoT Platform Design and Implementation

In order to improve aspects like the reliability of control and the process quality, Distributed Control Systems (DCS) have emerged. Industrial automation and control systems include many distributed systems that are often connected with each other over a network, therefore data produced in one system component must be shared with other components. Applications in these systems may have hard deadlines by which the data must be delivered, to process it on time to make critical decisions. Various middleware technologies have been proposed by international standardization organizations, industrial consortia, and research groups over the past two decades. These proposals aim to facilitate the communication of industrial control in a DCS and hide the heterogeneity between the subsystems. These technologies can simplify the design of the system and integrate control devices, even considering the heterogeneity of the components involved. Despite all the advances in the field of middleware technologies, there are several significant challenges in meeting the requirements of DCS systems [22]. As this project aims to be a customizable solution, capable of meeting the demands of the most different customers, the proposed solution will be called as a platform [23]. A platform is a unit that guarantees or supports the interoperability of its components according to certain rules or terms, that is, an environment capable of accommodating the specific requirements of each company, instead of middleware, a solution that does not allow customizations (the same solution for all customers).

A three-tier architecture pattern will be used to describe the platform, in order to facilitate its understanding. An architecture pattern is a simplified and abstracted view of a subset of an IIoT system implementation that is recurrent across many IIoT systems. Architecture patterns represent some common, typical, and essential features of IIoT implementations that are easy to recognize and understand by practitioners. Coherent IIoT system implementations follow these well-established architectural patterns [24].

**Figure 2:**
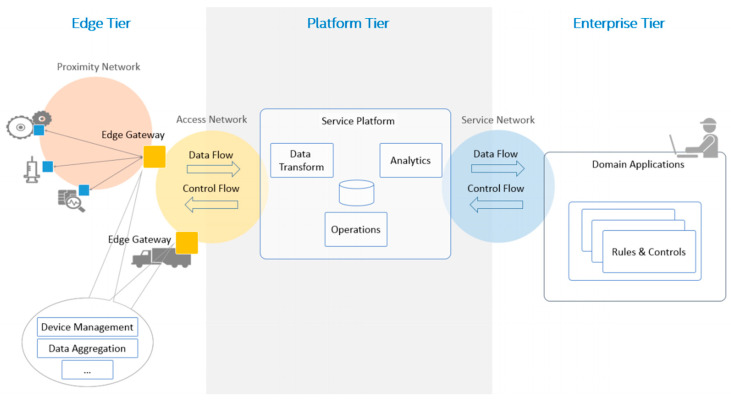
Three-tier architecture pattern. Source: [24].

The relationship between the three-tier architecture and the functional domains is presented in Figure 2. This relationship between the functional building blocks and the tiers is based on the main functionality or purpose of the tier but are not exclusively assigned to that tier [24]. The Edge tier represents the shop floor level that will be composed of a factory automation bus and the robotics systems for implementing Industry 4.0 architecture. In this sense, following the instructions of RAMI 4.0, to implement the information/communication layer, the open standards OPC-UA [25] was chosen. Based on the requirements of the EMBPT and TSK industrial pilot cases, the differentiation and demands for interoperability between the robotic section of the shop floor and the discrete/additive manufacturing section have been strongly supported. Therefore, it was decided to proceed with a separation from the robots (and their standards) and from the machinery/automation components (and their standards).

In the Platform Tier, there will be two lanes: one based on FIWARE [26], providing a set of interoperable components for managing events and data coming from complex manufacturing systems in a typical event-driven architecture. The other, based on APACHE [27], is popular and easy to interoperate with several existing manufacturing IoT systems. This layer will also contain the IoT Repository. In the Enterprise Tier will be the Analytics components and the Enterprise applications like ERP and MES.

**Figure 3:**
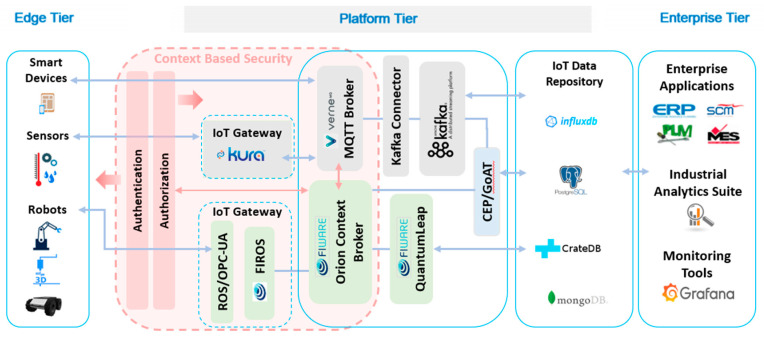
FASTEN Industrial Internet of Things (IIoT) platform.

As previously stated, the FASTEN IIoT platform was designed as an open platform and therefore can be customizable according to the requirements of the customers. The platform (Figure 3) was configured to meet the requirements of the two pilots of the project: the FIWARE (gray background color) lane was customized to the TSK pilot and the Apache lane (background color green) for the EMBPT pilot. Therefore, in situations where new features are required, they can be added to the platform. Likewise, if some features/tools are not used, they can be discarded. The flow of data in the system will be described below.

**Figure 4:**
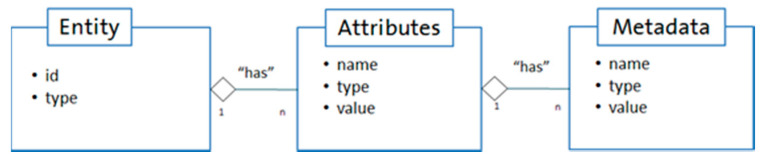
Main elements in the NGSI (Next Generation Service Interface) data model.

The blue box, shown on the left side of Figure 3, represents the Edge layer, where the devices, robots, and sensors are located. Considering the FIWARE lane (green boxes), data from the Edge tier will be sent to the IIoT Platform (in the Platform tier) using ROS (Robot Operating System) [28] or OPC-UA. If ROS is used, for example, data will be placed on a topic (like a queue) by ROS and will be retrieved by FIROS [29]. FIROS is a tool that enables communication with ROS, working as a translator between robotic systems and the cloud, transforming ROS messages into NGSI (Next Generation Service Interface) [26] to publish them into the FIWARE Orion Context Broker and vice versa. The main elements in the NGSI data model are context entities, attributes, and metadata, as shown in Figure 4. FIWARE NGSI is intended to manage the entire lifecycle of context information, including updates, queries, registrations, and subscriptions.

Thus, returning to the description of FIWARE lane data flow, context entities will be generated in the FIWARE Orion Context Broker, one for each device, which stores the information of the current state of each sensor. The attributes of these entities will be updated as messages from each device reach the IIoT platform, thus maintaining an image of the behavior of each device in operation. The current state of each sensor is saved in a streams database, in this case, CrateDB [30]. The connection between the Orion Context Broker and the database is made by the QuantumLeap application, which also belongs to the FIWARE framework. These data, when persisted, can be used for system monitoring, performed here by Grafana [31]. This step ends the FIWARE lane data flow.

The IIoT Platform also provides a device discovery and registration mechanism implemented through the Watchdog component of the FIROS tool. Watchdog receives notifications about new robots and robots that are no longer available. FIROS will create/remove entities in Context Broker according to these messages.

In the case of Apache lane (gray boxes), the devices can connect via Eclipse Kura [32] or directly to the MQTT Broker. Eclipse Kura is an extensible open source IoT Edge Framework based on Java/OSGi and offers API access to the hardware interfaces of IoT Gateways, like serial ports, GPIOs, I2C, etc. It features ready-to-use field protocols (including Modbus, OPC-UA, S7), an application container, and a web-based visual data flow programming to acquire data from the field, processes it at the edge, and publishes it to leading IoT Cloud Platforms through MQTT connectivity. In order to perform the function of MQTT Broker, the VerneMQ [33] tool was chosen. A connector continuously monitors the topics (like a message queue) in MQTT and as soon as a message arrives it is automatically transferred to the Apache Kafka [27] streaming platform. The data from Kafka is saved in a time series database, in this case, InfluxDB [34].

Following the sequence, the two lanes are connected to a blue box, representing complex event processing (CEP) and GoAT (Greatest of Actual Time) [35]. CEP is a set of tools and techniques for analyzing and controlling the complex series of interrelated events in distributed information systems allowing to understand what is happening within the system. It also makes it possible to identify and resolve problems quickly, allowing events to be used effectively to improve operation, performance, and security [36]. GoAT represents our broker proposal for sensor ranking.

After processing by our Sensor Ranking broker or other CEP services, messages is saved in the IoT Data Repository that has some DBMS: MongoDB is used by Orion; InfluxDB is used in Apache lane; CrateDB; PostgreSQL is used by an ERP system, involved in predictive tasks, which is in the Enterprise tier. The Data Repository is the last component of the Platform tier.

Enterprise applications, like ERP and MES, Analytics Suite and Grafana, a tool for monitoring the system, are located at the Enterprise layer.

Both FIWARE lane and Apache lane can run in a distributed way, allowing parallel and distributed processing, providing scalability to the systems involved.

Finally, in the IIoT Platform security can be provided by traditional methods, with features like authentication, identity management, and access control (FIWARE), and can also be enhanced using context-based security (CBS) [37,38]. In this case, the context is considered as a first-class security component to direct the behavior and permissions for each IoT device. This allows smart objects to be enabled with context-based security solutions, based on the premise that, as the context can change at any time, security decision making will adapt to the context in which transactions are performed.

## 3. Related Works

An IIoT platform requires data manipulation from heterogeneous devices (for example, robots and sensors) in industrial environments and acts as an intelligent data repository for other layers, such as the optimization and forecasting layers. This allows the improvement of service quality and, at the same time, meets the requirements of Industry 4.0. In these environments, a large amount of hardware and software elements involved generating a huge amount of data in the most diverse formats. As environmental requirements, these data must be processed in a restricted time and evaluated due to the uncertainty inherent in those environments.

However, several challenges stand between the conceptual idea of IIoT and the full deployment of its applications. A well-defined, scalable, backward compatible, and secure architecture is required to bring the IIoT concept closer to reality. The platform requirements, listed in Section 2.2, will be used to compare the different approaches of the related works, more specifically those focused on platforms and middleware for Industry 4.0.

The work of [39] presents an Agent-based Complex Network Architecture (ACONA) structure. The main objective of the framework is to meet the demands of cognitive architectures in industrial systems. Agent functions natively offer multiple common development patterns, reducing the time required to implement the infrastructure. One of the main features of the framework is to offer flexibility in the design of controller systems, simulations, and cognitive architectures. It also allows the construction of different types of modular systems and network topologies.

The authors of [40] developed an online/offline Data Sharing Framework (DSF) for Cloud-Assisted in IIoT environments, which supports online/offline encryption, outsourced decryption, and constant trapdoor (or data user secret key) generation capabilities. They demonstrate that the DSF is selectively secure in the chosen access structure security model and demonstrate its efficiency and feasibility in practical scenarios.

The authors of [20] present VICKI, an approach in which the general objective is to provide new mechanisms for notifying registered services with information produced at the factory. Combining technologies like IIoT, Semantic Web, and systems based on multiple agents, the proposed structure allows resources to be updated on the changes that have occurred in their context. Thus, the structure can be explored in scenarios where cooperation between resources is an essential requirement. The framework presents a solution that offers resources for connectivity, publish/subscribe (typical services of IoT applications), persistence, scalability, real-time, and reasoning about data coming from the IoT environment. Despite this, it does not offer solutions to guarantee the safety of the environment and it also does not offer flexibility to carry out customizations in the solution.

The authors of [41] focus on the integration and interoperability across different platforms, through dynamic and reconfigurable solutions for discovery and integration of data and services and also considering a privacy-aware and secure solution for crawling, indexing, and searching in distributed IoT systems. The proposal presents solutions for the environment of Industry 4.0 among others. This is the only related work that claims to offer security solutions, although the authors do not describe how this is done and do not show results.

In [42], the authors present Cilia, a mediation middleware designed for smart manufacturing based on service-oriented components. In this middleware, an integration solution is a composition of domain-specific components called mediators. A mediator implements a single mediation operation and is structured into three elements to deal with synchronization, processing, and routing issues in a modular way. Specific mediators, called adapters, implement communication protocols and handle the dynamicity of resources. Mediators are connected via bindings. A binding describes a connection between an output port and an input port. At execution time, it is realized by a communication protocol transferring data from one mediator to another. This work has as one of its advantages the flexibility in the configuration of the services, however, it does not allow the use of the framework in a distributed way, which compromises the scalability of the model. In addition, it also does not offer features to ensure data security.

DAQ-Middleware [43] is located in the acquisition layer, considering an IoT architecture hierarchy model with four layers: sensing, acquisition, management, and application layer. Its functions include receiving control commands from the application system, obtaining data from data sources, and transmitting them to the application system through the standardized data interfaces. The main functions of the work are to design the middleware structure model, with a parallel data acquisition algorithm, and realize the automatic access to different data sources, with the goals of reducing the development cost and improving the data acquisition efficiency. The framework, however, does not offer real-time processing and security features.

Perera et al. [44] propose an IoT middleware solution that can be run with MapReduce [45] based techniques to increase the scalability of the solution. They developed an ontology-based context framework for sensors in IoT which allows capturing and modeling context properties related to sensors. This information allows users to search the sensors based on context. In order to index and rank the sensors, the user preferences are used with an indexing technique based on weighted Euclidean distance.

Table 1 illustrates a summary of the characteristics of each of the proposals discussed above. The symbol “√” represents the presence of the feature in the proposal while the symbol “-” represents the absence. The column “Interoperability—Message Patterns” uses two codes: PS to publish/subscribe and RR for request/response. The “Resources Control” column uses DIRS encoding that represents the initials of the discovery, indexing, ranking, and selection of sensor functions. The column “Interoperability—Application Protocols” presents the name of the technology used. The column “Persistence” presents what kind of database manager is used: RDBMS, NoSQL, NewSQL, and ALL if all DBMS are accepted.

Analyzing Table 1 (disregarding the proposal of this work) itis possible to verify that, among the proposals presented, with respect to security, only the IoTCrawler and DSF solutions have this feature. Another frequent problem in the proposals analyzed is related to the persistence of data: either it is absent in the solution or it has limitations, as is the case of the CASSARAM proposal, which works only with relational databases. Another aspect to be considered is that only one proposal has all the resources for the control of sensors, that is, discovery, indexing, ranking, and selection.

## 4. Experiments

This section describes the use of the platform in scenarios created in each of the pilots.

In the case of TSK, the environment of an additive manufacturing unit assembled in a laboratory will be presented. This case demonstrates the use of the platform in a real TSK pilot case. This use case meets some of the requirements listed in Section 2.2, as follows: the scenario described in the use case meets the interoperability requirement when connecting different devices, more specifically, two robots (manipulation and navigation) and a 3D printer. The resource control requirement is demonstrated using context entities created to reflect the state of each device in the Orion context manager. The real-time aspect is met by demonstrating the monitoring of the state of the environment in the Grafana tool and, also in a non-explicit way, when the system allows the customer to be informed about the start of production of the order, in addition to being able to follow updates on general progress and possible delays. This can be done through the mechanisms of publishing and subscribing. In this use case, data persistence is performed by the CrateDB manager. The other requirements of the platform, which are not explicit in the example, will be demonstrated in the section of scientific contributions of the work.

In the use case of EMBPT pilot, two scenarios are described, one simulated and the other real. The first situation describes the monitoring of temperature sensors in an environment (simulated). The scenario for the second example describes the use case for a pick-and-place robot. The environment was set up at the Embraer Évora factory, in Portugal, in the area of Logistics Automated Warehouse. In the final subsection, data regarding the IIoT platform performance tests are presented.

In this use case, it is possible to identify the following platform requirements: the scenario described in the use case meets the interoperability requirement when handling pick-and-place and navigation robots. The resource control requirement is met using CEP services. The real-time aspect is also demonstrated by monitoring at Grafana and by the customer’s interaction with the various stages of production. Data persistence is performed by the InfluxDB manager. As in the TSK case, the other requirements are discussed in the scientific contributions section.

### 4.1. Thyssenkrupp Use Case

**Figure 5:**
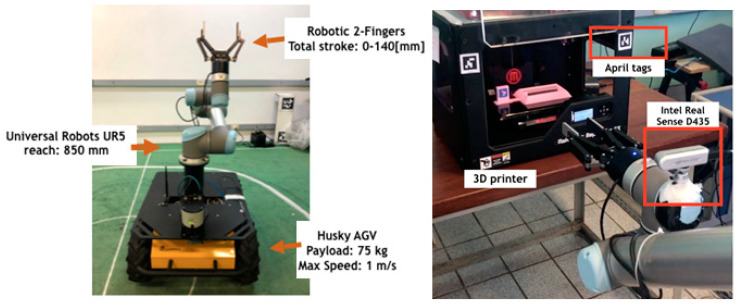
ThyssenKrupp (TSK) use case: mobile manipulator robot (**left**) and 3D printer in the laboratory’s Smart Robotic Additive Manufacturing (SRAM) unit (**right**).

**Figure 6:**
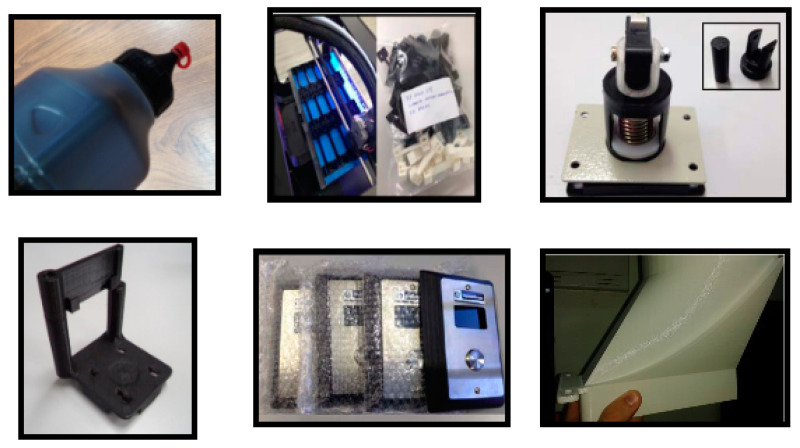
Different 3D parts currently printed through additive manufacturing.

The Smart Robotic Additive Manufacturing unit (SRAM) of the TSK use case, consists of a unit equipped with a 3D printer and a mobile manipulator robot. The mobile robot consists of a robotic manipulator arm mounted on an Automated Guided Vehicle (AGV). The robotic platform is equipped with laser scanners and visual sensors (cameras), providing data to be used for the autonomous location and navigation (Figure 5). In Figure 6 it is possible to see different parts printed in the process.

**Figure 7:**
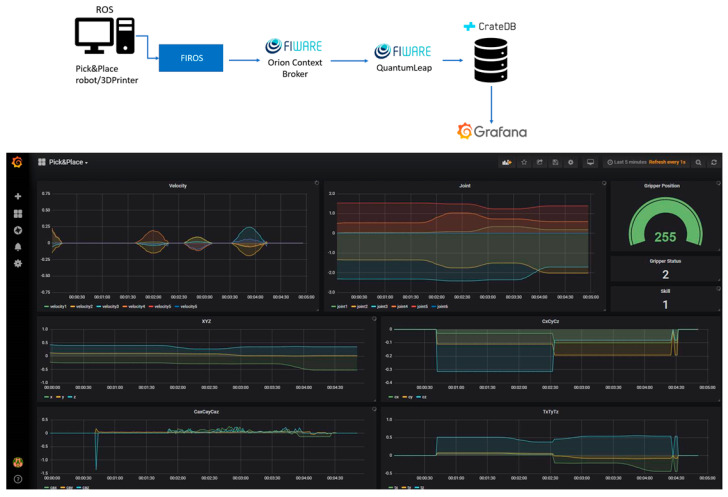
TSK use case: FIWARE architecture (**top**) and a dashboard for pick and place task (**bottom**).

The FIWARE lane architecture and a panel showing the status of a picking order are shown in Figure 7. In this example, the data comes from the 3D printer via ROS. They are read and sent to the Orion Context Broker by FIROS (FIROS works as a translator between robots and the world of the cloud, transforming ROS messages into NGSI to publish them in the cloud and vice versa).

**Figure 8:**
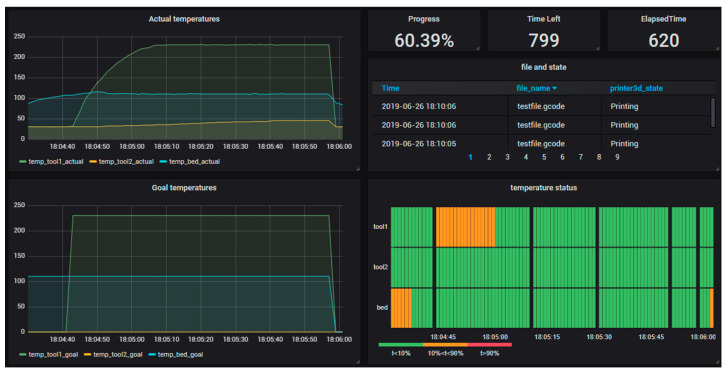
Monitoring 3D printer status and printing process.

What FIROS does is request that Orion updates a context entity related to the device in use with the new values that reflect the current state of the device, in this case, a 3D printer. This context entity has information about the part being printed, as well as information collected by the printer’s sensors. The context entity for the printer used in the test was previously configured to be saved in the CrateDB database. This allows to keep track of changes and create processes to monitor device usage and print environment information, like the temperature of different printer components. The monitoring of the printing process in the Grafana tool can be seen in Figure 8.

### 4.2. Embraer Use Cases

In the case of the EMBPT pilot, two use cases will be described. The first simulates temperature monitoring in an environment and will be useful to describe the flow of information on the IIoT platform, as well as the use of complex event processing (CEP). The second describes the scenario of automated parts handling in the company’s warehouses.

CEP rules implemented via Kafka Streams take their data from Kafka topics and write to (usually different) Kafka topics. They work inside the Kafka framework, transparent to downstream consumers who do not have to know who or how the data was processed. Any part of the system that wants to use these CEP results can just listen to the produced Kafka topics, without being coupled to the way the data was processed. Additionally, those independent CEP rules can use each other’s results for further processing to build up more complex CEP chains.

In this use case, the IoT devices (temperature sensors) send captured data to the IIoT platform. Once an improper temperature value is detected for an environment, the event handlers generate a new message for some process responsible for activating the environment maintenance activities. Likewise, any other type of failure that may occur can be addressed through actions of control and maintenance processes. There are many ways and frameworks available to do CEP with the FASTEN IIoT Platform. For example, there are Kafka consumer packages for Apache Spark and Apache Flink. To connect these, just connect them to the running Kafka broker.

**Figure 9:**
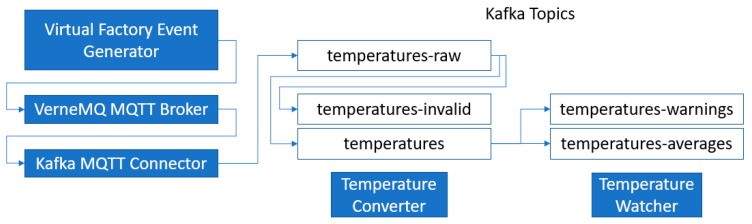
Temperature monitoring example.

This example, in which Figure 9 shows the respective data flow, presents a showcase for another kind of CEP framework using the relatively new Kafka Streaming API. In this case, CEP rules are run by small, independent, and stateless Java processes. Two of such CEP rules are used in this test: the first CEP rule checks the JSON structure of the payload and only forwards “valid” temperature readings. It also converts the different temperature units into °C. The second CEP rules generate messages for two things: “Warning” events are generated if temperatures get too high depending on the “location” of the measurement, and averages are computed to be able to combine high frequency measurements into low frequency temperature events.

**Figure 10:**
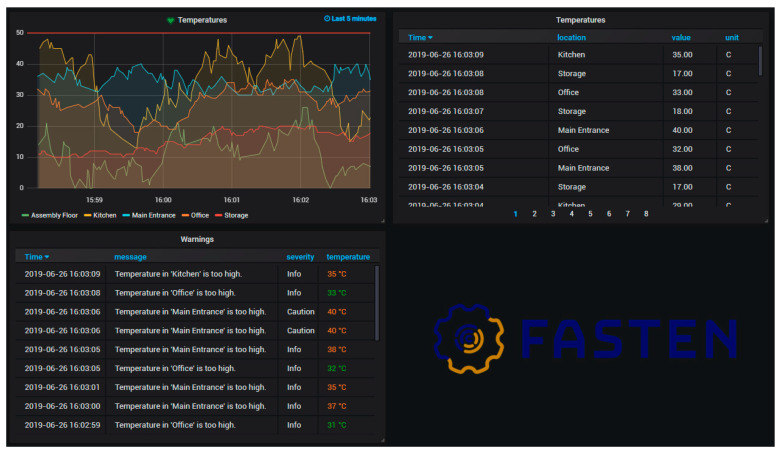
Monitoring temperatures in Grafana.

The idea is to be able to take work away from downstream data processing by doing it once in this highly scalable infrastructure. Figure 10 shows the Grafana tool monitoring script for this use case.

**Figure 11:**
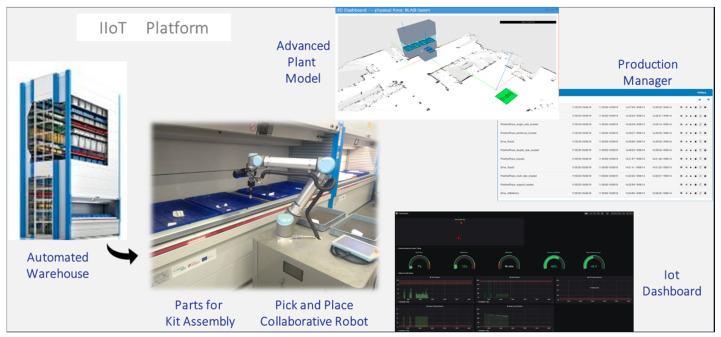
Main components of the adaptive pick and place robot in the logistics warehouse.

The demonstration of the second use case, Adaptive Pick and Place Robot, consists of the provision of baskets (the blue boxes in Figure 11) by the Automated Warehouse, which contains the parts that are required in the Wing Assembly Line. A kitting order from the Warehouse Management System, specifying these parts and their location in the boxes is emulated and sent to the Advanced Plant Model. In this time, an appropriate plan of action (production scheduling) is generated. This is retrieved by the Production Manager, which starts executing and monitoring the plan, by requesting the execution of navigations and pick and place tasks from the mobile platform. The robotic platform moves into the Automated Warehouse and starts picking the parts from the blue baskets and placing them in the two kitting boxes that the platform carries. In the end, the robotic platform moves to a transfer point in the logistics area where the two kits are retrieved by human operators and transported into the wing assembly line.

**Figure 12:**
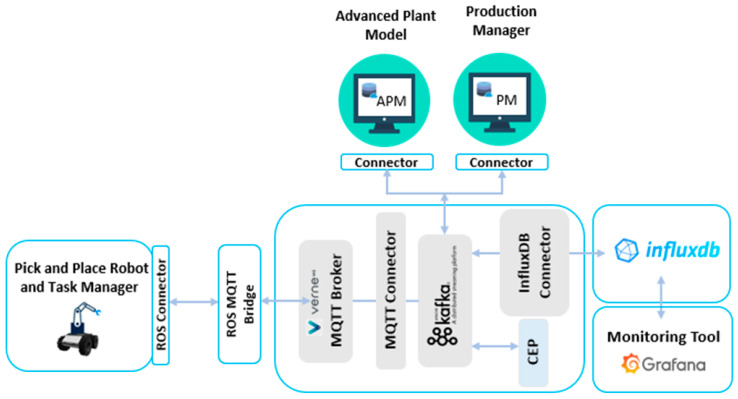
IIoT platform: Apache lane integrating the Task Manager (pick and place robot), the Advanced Plant Model, and the Production Manager.

Behind the visible things, the IIoT Kafka lane (Figure 12) is running in the back end and supporting all communication between the Advanced Plant Model, the Production Manager, and the Task Manager (the software component embedded in the robotic platform). A real-time dashboard provides data about key functions in the robotic platform (e.g., location in space, the strength of Wi-Fi power along with space, CPU and memory load, battery energy level). The operational status of the robot as it executes the plan of action is shown by the Production Manager and Advanced Plant Model.

### 4.3. IIoT Platform Performance Test

Two versions of the IIoT reference architecture were implemented in FASTEN, which can be used by customers according to their requirements. Next to the availability of interfaces, scalability, latency and robustness are criteria to decide which of the platforms meet the use case requirements best. In terms of scalability and robustness, all core components of the Apache lane were designed to fulfill these requirements. In addition, using additionally container orchestration frameworks provided by Docker Swarm or Kubernetes would ensure that each traffic will be processed at every time by starting the right number of instances, doing some load balance for adjustments, and restarting instances in case of failures. The same considerations can be made for the FIWARE lane.

However, the orchestration of the container was not in the scope of this research project and it was shown that the developed solution will provide more than enough power to meet all performance and robustness requirements of the EMBPT and TSK use case scenarios.

**Figure 13:**
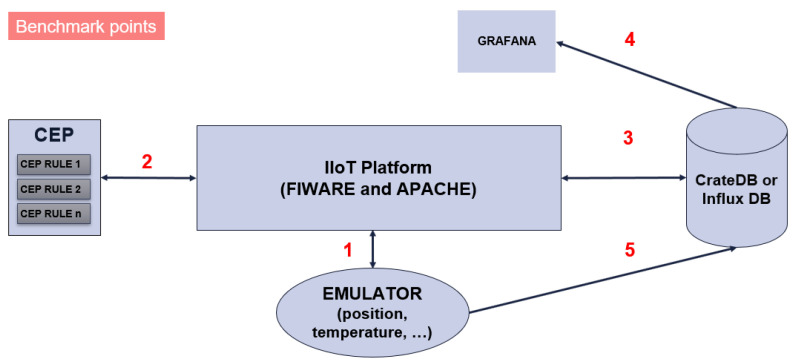
Measurement of data transmission time between the elements of the IIoT platform.

Nevertheless, a concept for measuring the data exchange times between the main components of both IIoT lanes was developed (Figure 13).

The data exchange times will be measured across five connections:

1. The time spent in both directions between a program emulating the event source and the IIoT platform.
2. The time spent in both directions between the CEP module and IIoT platform.
3. The time spent in both directions between the database and the IIoT platform.
4. The time spent between the database and Grafana.
5. The time spent from the emulator program to the database.

**Figure 14:**
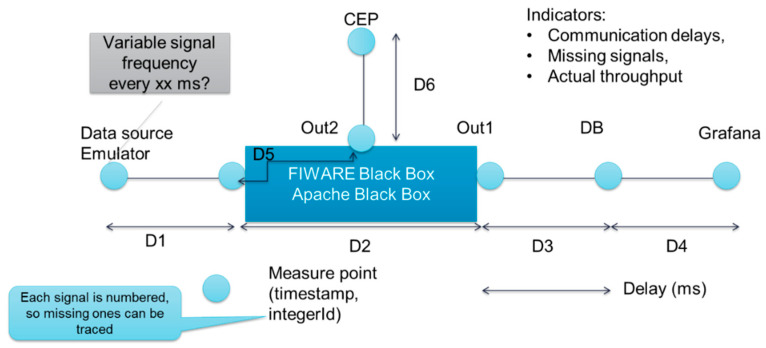
Measured connection in the IIoT platform.

Figure 14 shows the data exchange connections where time measuring will be conducted in a more schematic manner. For doing this, data stamps were added to the data when it was leaving one component and when it was reaching the other component. The subtraction of both times leads to the time to be measured.

The time is measured in milliseconds for the following connections:

- D1: Event simulator and IIoT platform.
- D2: IIoT platform main entrance and IIoT platform database out.
- D3: IIoT platform database out and database (DB).
- D4: Database and Grafana.
- D5: IIoT platform main entrance and IIoT platform CEP out.
- D6: IIoT platform CEP out and CEP module.

Three indicators will be measured to compare both lanes:

- Communication delays:
  The pilots defined as a time requirement for use cases that an event could not take more than 1 s between the generation and viewing of the event on the monitoring panel. In order to ensure this performance, a threshold of 50 ms was defined for each data transfer (connection).
- Actual Throughput:
  Number of events.
- Lost Events:
  There is no requirement coming from the use cases related to the quality of service. Additionally, it is difficult to define a general threshold. If the same value is coming several times a second, like temperature, it is no problem to achieve 98% of successfully delivered events. However, if it is a steering event coming from the APM, none should be lost.

This was the starting point for implementing the set of tests and the results analyzed in terms of time and productivity.

**Figure 15:**
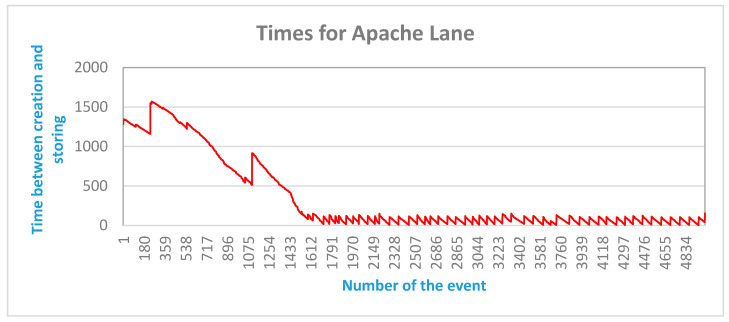
Apache lane 5000 events and eight cores: time of event creation and saving.

The diagram presented in Figure 15 leads to the following results:

- In the first second after starting the test run, around 1000 events were created and the time between creation and storing each of them in the database was 1.3 s in the beginning and 1 s in the end.
- In the next second after starting the test run, again around 1000 events were created and the time was between creation and storing them in the database was 1000 ms in the beginning and 500 ms in the end.
- Then the time was decreasing to a stable range between 200 ms and 40 ms.

These results show that after 2 s of running the Apache IIoT lane, the answering time was far below the requirement of 1 s and that the system is able to process around 1000 events per second.

**Figure 16:**
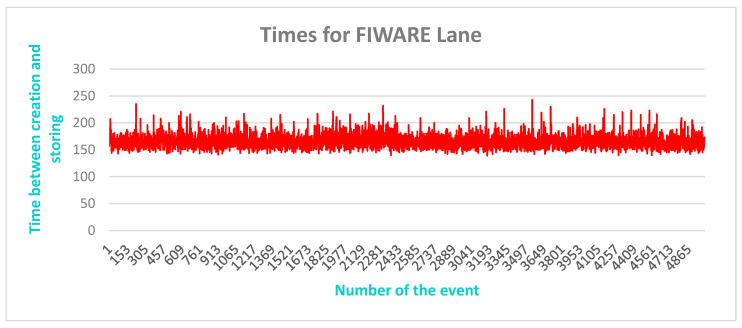
FIWARE lane 5000 events and eight cores: time of event creation and saving.

Comparable results of a test for the FIWARE lane are shown in Figure 16. In this test series, 5000 events were created by the event generator and saved in the database. The diagram shows the ongoing time between the start and end of the series. As shown in the image, the average time was in the range between 150 ms and 250 ms, demonstrating that the FIWARE lane also meets the time requirements.

## 5. Discussion

In this section, the scientific contributions of the work are discussed, as well as the contribution to the pilots. It also includes a discussion of aspects related to the real-time nature of the platform and its impact on each of the pilots.

### 5.1. IIoT Platform Real-Time Approach

Taking advantage of the vertical and horizontal integration, FASTEN will reduce the variability caused by a diversified and personalized demand, implementing a scalable and modular system for additive manufacturing processes, which is flexible and able to adapt to new requirements. Ensuring real-time coordination between production and logistics activities allows real-time decision making to be more systematic and confident, based on predictive and prescriptive analysis of the manufacturing system. Developing a global responsive system, with the adoption of a standardized data repository and decision-making integration throughout the supply chain, FASTEN IIoT will lead to the empowerment of businesses (with an integrated knowledge platform that continuously provides real-time information on the state of the manufacturing system) and end-users (using a simulation environment that engages stakeholders in the product design, allowing them to perform simulations and forecasts).

In custom-product design, the customer is a direct intervenient for he takes part in the design and configuration of the product. In manufacturing, assembly, and packing, the real-time information flow is mostly unidirectional. The customer is informed of when their order has started production and can track the updates on overall progress and any possible delays. Additionally, he can be given an estimate of the predicted time of completion, and all this helps to increase the transparency between the production processes, i.e., the real-time situation of the system, and the customer [46,47].

Intelligent/advanced sensors and robots allow faster and more efficient information flow and system reconfiguration, reducing costs and lead times. The high volume of data generated by these elements will also support, for example, component health and condition monitoring of critical components, allowing a reduction of machine failures and helping to adopt preventive maintenance measures, thus increasing system uptime and availability. The IIoT platform connects all elements, working as the middle layer that coordinates the whole framework. The data from sensors, customers, suppliers, etc., are used to manage production and assist in real-time decision-making. Moreover, customer behavior, order patterns, production cycles, and many other predictive inferences can be made with resource to the data that are exchanged and obtained by all the components of the value chain. These forecasts are also inputted in simulation-optimization models to plan and control production ahead of time and understand the impact of specific decisions. Data from the production site and the whole value chain are also useful to monitor and assess both historical and real-time system performance, as well as provide prescriptive insights on the system.

In particular, EMBPT will explore the full integration of the IIoT platform into their production systems: some assembly operations require the preparation of parts that must be stored and managed in large, complex warehouses. Full incorporation of all stored items into the IIoT will provide the necessary information for the predictive and prescriptive real-time simulation-optimization to track these items and to assist the system in directing the multipurpose robots, for optimized storing and retrieval. This will be done using advanced machine learning and real-time plant monitoring, without the requirement for rigid indexing of moving parts inside the warehouses.

In the TSK case, the system will provide a proper environment to develop and explore fully integrated additive manufacturing capabilities, powered by the IIoT close connection to the remaining system elements, providing immediate part availability and improving maintenance operations.

Both pilot use cases will explore the benefits of standardized components and architectures that offer the possibility of fully modular and scalable manufacturing systems. The resulting highly advanced systems will be able to provide the real-time flexibility and responsiveness required to achieve “lot size one” manufacturing, with maximum levels of productivity.

### 5.2. Contributions to Pilot Cases

One of the objectives of FASTEN IIoT is to significantly reduce the time that goes from custom-designed orders, the order execution, and the delivery of the request itself. Concerning the IoT use cases, at this point, TSK is primarily looking to reduce the time it takes to provide maintenance to their customers. This involves ordering and production stages for custom-designed parts, required for specific repairs, as well as the lead time required for getting those parts to the customer.

On the other hand, the main objective of EMBPT is to become more efficient in its warehouse operations. The objective here is to increase productivity levels in relation to the storage, location, and retrieval of a wide variety of parts in a fully automated system. This will reduce the time required to supply materials to the machines in operation, allowing a significant increase in productivity. In addition, it will also be possible to obtain a more flexible production line, aiming at a faster adaptation to changes in design requirements, without affecting the production flow.

These results are achieved using application software for predictive and prescriptive analysis of manufacturing systems (robots and additive manufacturing units) and for digital manufacturing services. This application includes a suite of robust simulation and optimization models and a platform for real-time monitoring and control of devices, automation systems, additive manufacturing units, and services for custom-designed products. In addition, the FASTEN framework also aims to accelerate the integration between product design and manufacturing, effectively controlling and assessing the performance of the whole manufacturing systems. This integration will be achieved by combining simulation, optimization, and analytics tools, to virtualize the whole system, thus providing a high degree of flexibility and agile decision-making in real-time. The adopted technologies will effectively approximate customers to products, and significantly reduce the global operational costs, leading to low unitary costs of custom-designed products, even when ordered in small quantities.

All these software components will use data captured from IoT devices and homogeneously stored in the databases used by the platform.

In this context, predictive model-based tools based on state-of-the-art algorithms and techniques will be used to reconfigure and adapt robotic and additive manufacturing technologies to fast-changing demands.

Moreover, to address large-scale and complex dynamic planning and scheduling problems, hybrid simulation-optimization techniques will be applied. With these techniques, it will be possible to handle complex systems that are computationally very demanding and cannot, in general, be managed by standard optimization algorithms. After that, process monitoring and target prediction will also be addressed, to trigger security alarms or to anticipate unpredictable system behaviors.

### 5.3. Scientific Contribution to the Manufacturing Industry

This section lists the scientific contributions of the work considering the most relevant topics for the research area.

#### 5.3.1. Distributivity

Global supply and distributed processes are always associated with considerable coordination and management time and effort. Industry 4.0 should allow information to cover long distances in near real-time. International companies will, therefore, be able to react quickly to customer requirements in globally distributed production systems, as well as provide their customers with a current picture of production progress at all times [48]. FASTEN aims to develop a framework that enables low-cost flexible manufacturing, thus achieving a profitable mass-customization system. The IoT cloud-based platform enables decentralized production, and the difficulties introduced by small batch production (expected when dealing with customized customer demands) will be overcome with the aid of predictive simulation tools to allow reconfiguring and adapting the manufacturing system to changing user requirements. The reference model RAMI4.0 will help frame and standardize the collection, processing, and exchange of information, and the adoption of existing protocols and standards. For example, within the value stream and the enterprise/process control systems, it will ensure viable integrations with other components (other MES/ERP systems) and ultimately accelerate the adoption of this technology within the industry.

#### 5.3.2. Scalability

The data generated by the IoT far exceeds the capabilities of existing companies’ IT architectures and infrastructures, and its real-time requirement will also greatly emphasize the computing power available. Taking into account the heterogeneity, scalability, real-time, complexity, and privacy of big data, data must be “extracted” at different levels during analysis, modeling, visualization, and forecasting, in order to reveal its intrinsic property, and thus improving decision making [49]. The cloud computing model is an efficient alternative for data management, contributing to greater predictability, allowing greater use without degrading performance [50].

However, exploring this technology from a mass scale perspective in a way that is cost-efficient requires significant consideration. FASTEN explores a close connection between flexible robot systems and the Predictive Real-time Simulation and Optimization system, linked through a Unified IoT Cloud platform. Thus, with the application of state-of-the-art optimization algorithms and meta-heuristics backed by complex scenario simulations, will result in reconfigurable and scalable additive manufacturing cells that support the mass-customization trend, where proper integration between product design and effective control of the production system’s performance will lead to a seamless adjustment of the manufacturing system, to changing user requirements and various other circumstances. Due to the real-time integration of data from the Robot and Manufacturing System and the IoT Cloud Platform, discard of outdated data and integration of specified user requirements, the suite will aid in the manufacturing system improvement, guaranteeing adequate delivery and response, according to the actual system state. This is achieved through informed data-driven decision making, incurring significant cost savings.

#### 5.3.3. Security

All component integration, including software, hardware, and systems, can be vulnerable to attack. As a result, a heterogeneous and complex environment leads to significant difficulties in ensuring the security and privacy of users, data, and systems [6]. In this sense, the context-based security can be defined as “a set of information collected from the user’s environment and the application environment and that is relevant to the security infrastructure of both the user and the application.” [37,38]. For example, while detecting an intrusion during communication, the security mechanism may adapt to a strong authentication method. Context should be a first-class security component to drive the behavior of IoT devices. This would allow smart objects to be enabled with context-based security solutions, to make security decisions adaptive to the context in which transactions are performed. At the same time, context information should be managed by considering security and privacy considerations.

The context-based security module allows the creation of rules that determine, pre-authentication, whether and how a given authentication process should proceed based on context. Examples of context information are (a) device registration and fingerprinting, (b) source IP reputation data, (c) comparing user’s current information with the corresponding information kept in a directory or user store, (d) geo-location, (e) geo-fencing, (f) geo-velocity, and (g) behavioral analysis [51,52].

There are two modules for context-based security: Context Provider and Context-Based Security. The Context Provider has the main function to produce context information from the users’ data. It acquires information about the users’ environment and behavior. Thus, this information alongside the one sensed by the FIWARE ORION Context Broker is the input for the Context-Based Security module. The FIWARE Security module provides authentication, identity and access management, and access control by the traditional methods. The Context-Based Security module provides dynamic security decisions by using context information. This module is based on the premise that the context may change over time, so the security mechanisms may adapt to the context. The core operation to provide context-based security is by using pre-defined security rules for the different situations that the context may change. These rules are mostly defined for a specific domain that the module is deployed in. It works in the follows steps: (i) it receives and analyzes the context information, (ii) matches the received context with the historical one, and (iii) infers security decisions by the rules.

#### 5.3.4. Interoperability

One of the requirements in these environments is to provide interoperability between robotic and manufacturing equipment through information exchange using standard protocols, and vertical connectivity with the IoT platform. The task of integrating robotic and manufacturing equipment of different purposes, architectures, and paradigms is still a major risk in automation projects, due to the variance of physical bus, communication protocols, and operating systems [17]. This situation often leads to system integrators typically specializing in a single vendor to ensure compliance of solutions and building expertise. As such, solutions of that kind are either forced to stay within the product family of a single vendor, or to develop the necessary interconnection between equipment. Recently, efforts have been made to propose a unified integration platform that interconnects robotic hardware and automation components from different manufacturers. The most significant is the OPC-Unified Architecture (UA) standard, backed by the OPC Foundation, an industry-backed initiative to enhance the interoperability between automation components by providing standard interfaces to MES, ERP, and HMI. The FASTEN Industrial IoT Platform will provide the interoperability between product and production system life cycle via a message broker and an enterprise service bus. The CPS-design and OPC-UA interoperability components are derivative work inspired by the CRYSTAL [53] Reference Platform/Interoperability Specification and subsequent CPS projects for safety critical embedded systems.

#### 5.3.5. Persistence

With many things connected to the Internet, a huge amount of data in real-time will be produced automatically by the connected things making data analysis a fundamental task in the Industry 4.0 environment. Building systems into which big data from a variety of heterogeneous sources are integrated can be a challenging task [17,54,55]. In this way, existing standards for lifecycle management and value flow (IEC 62890), for the integration of corporate control systems (IEC 62264, ANSI/ISA-95) and for process control (IEC 612512, ANSI/ISA-88) provided a solid foundation for the development of the platform and also ensured the required compliance, accelerating the adoption of this technology by industrial companies. As a result of the platform’s connectivity requirements, a semantic repository was created, supported by a common reference data model, capable of guaranteeing digital continuity throughout the site and throughout the product’s life cycle. In addition, the semantic repository can structure, integrate, and interpret information, ensuring that the relationships between the characteristics of the entities can be coherently defined and used. The ICT platform is based on existing open source IoT platforms recently developed in P&I programs, and its main functions are provided through the integration of an event-oriented and service-oriented architecture.

#### 5.3.6. Real-Time

Traditional real-time performance guarantees are insufficient for CPS when systems are large and spatially, temporally, or hierarchically distributed in configurations that may rapidly change. With the greater autonomy and cooperation possible with CPS, greater assurances of safety, security, scalability, and reliability are demanded [3]. In order to standardize the data repository and the integration of decision making, from the services of end users (consumers) to the levels of manufacturing and supply, these objectives were achieved through the review, analysis, extension, and testing of the existing integration standards. The adoption of the RAMI 4.0 reference model frames and supports the development of point-to-point connectivity and a standard system. The use of data stored in the platform’s persistence layer allowed the integration of product design and manufacturing processes, combining simulation, optimization, and analysis tools, providing a real-time decision-making process.

## 6. Conclusions

This paper presented the FASTEN IIoT platform, an EU-Brazil collaboration project funded by Horizon 2020 EU program. The main contribution was to improve reliability in the control and quality of the process in industrial environments, offering an open and customizable platform to meet the requirements of Industry 4.0.

In the IoT environment, data manipulated is complex and heterogeneous. As a result, they have characteristics of big data in aspects related to volume, velocity, variety, veracity, and vulnerability.

In an automated industrial environment, there is an enormous amount of hardware and software elements generating data continuously, resulting in a large amount of data that must be processed in a restricted time (volume and velocity). The presented platform can manipulate this data and devices using software components capable of processing data in a parallel, distributed, and time-restricted manner.

In addition, the variety of objects involved directly implies how these devices send and receive data (variety). The presented solution manipulates the data generated by these devices using a flexible set of communication protocols.

Another feature present in the IIoT Platform is related to the ability to deal with the inaccuracy of data from the devices involved (veracity). Thus, the platform presents methods capable of dealing with the uncertainty inherent in data obtained from sensors, through the use of CEP methods.

Finally, to meet the security requirement, preventing attacks and failures, it is necessary to monitor devices in real-time (vulnerability). For this, in the IIoT Platform, security can be provided by traditional methods, such as authentication, identity management, and access control, and can also be enhanced using context-based security (CBS).

In parallel, it was demonstrated how the IIoT Platform overcomes the challenges imposed by the growing demand for customized manufacturing. Examples were products with shorter life cycles, low volumes per order, and increase in the number of devices. It is also important to highlight the increase in intelligence in these devices, as well as distributed manufacturing. All these new scenarios are creating challenges related to technologies for connecting and integrating smart devices, managing access control and distributed and reactive computing in the IIoT environment.

Therefore, manufacturing companies require flexible solutions, capable of efficiently manufacturing and delivering customized products quickly. Likewise, it is necessary to rely on technologies to improve the connectivity of smart devices and platforms that can easily integrate and manage a potentially large number of smart devices, capable of efficiently collecting and managing data from these devices. Many of these features have been demonstrated using the IIoT platform in real scenarios of Thyssenkrupp and Embraer pilots. The other requirements that the platform proposes to fulfill, and which are not so clear in the use cases, are presented in a theoretical way in the section of scientific contributions.

Thus, the FASTEN IIoT Platform seeks to provide solutions to the above problems, managing data and information safely and quickly and making that data available to other layers of software involved in the smart manufacturing process.

On the other hand, analyzing the existing proposals, it is possible to verify that most of them offer resources to overcome only part of these challenges. Through a review of the literature, it was possible to notice that most do not have the resources to provide system security. Another aspect concerns to the persistence of the data: it is missing from the solution or has limitations. We can also highlight that only two proposals have all the resources for the control of sensors, that is, discovery, indexing, ranking, and query. Finally, it should be noted that most of the proposals studied do not offer flexible solutions, which can be easily adapted to the requirements of each customer.

Although the project is still under development, scenarios of its use have already been presented, with real data and simulated data.

As future work, tests of Complex Event Processing (CEP) are foreseen to control the operational environment through the treatment of data subsets. This is a mechanism that facilitates immediate feedback to the operating environment, optimizing the use of resources and adding security to the environment. Two ways of using the CEP methods are foreseen: one for using a sensor ranking method, which through the analysis of the data produced by each sensor will rank them according to the reliability of each one. The second form of use consists of an improvement of the process described in the case of simulated use of the EMBPT pilot, more specifically the one that demonstrates the temperature control in the environments. This approach will be used to validate the reliability assessment provided by the ranking method. The development of a mechanism that will use blockchain technology to guarantee authenticity, integrity, and security of access is also planned. In this case, smart contracts will be used to monitor the messages exchanged between the devices and the platform, to ensure that the requirements related to information security are met.

## Figures and Tables

**Table 1:** Features of related works.

Name	Distributed	Interoperability Application Protocols	Interoperability Message Patterns	Scalability	Security	Resources Control	Flexibility	Real-Time	Persistence
ACONA	√	MQTT	PS	√	-	-I-S	√	-	NoSQL
DSF	√	-	RR	-	√	---S	-	-	-
VICKI	√	MQTT	PS	√	-	-I-S	-	√	NoSQL
IoTCrawler	√	MQTT	PS	√	√	DIRS	√	√	-
CILIA	-	OPC-UA, REST	PS	-	-	-I--	√	√	RDBMS
DAQ-Middleware	√	MQTT, REST, FTP	RR	√	-	-I--S	√	-	-
CASSARAM	√	-	PS	√	-	-IRS	-	√	RDBMS
FASTEN IIoT	√	OPC-UA, MQTT, REST, …	PS	√	√	DIRS	√	√	ALL
